# Spatial and temporal patterns of a pulsed resource dynamically drive the distribution of specialist herbivores

**DOI:** 10.1038/s41598-019-54297-6

**Published:** 2019-11-28

**Authors:** Violette Doublet, Cindy Gidoin, François Lefèvre, Thomas Boivin

**Affiliations:** 1 UR 629 Recherches Forestières Méditerranéennes, INRA, 84 914 Avignon, Cedex, 09 France; 2 Department of Evolutionary Biology and Environmental Studies, University of Zurich, Winterthurerstrasse 190, CH-8057 Zurich, Switzerland

**Keywords:** Forest ecology, Entomology

## Abstract

Patterns and drivers of the spatio-temporal distribution of herbivores are key elements of their ecological and evolutionary impacts on plant populations. Herbivore spatial distributions may be influenced by increased (RCH: resource concentration hypothesis) or decreased (RDH: resource dilution hypothesis) resource densities, but the effect of temporal variations in resource densities on such distributions remains poorly documented. We used a survey of a masting tree species and its seed predators in Southeastern France to address the effect of a host’s pulsed resource on the spatio-temporal distributions of highly specialized insect herbivores feeding on seeds. Variations in both resource and seed predator densities were assessed by estimating seed production and seed infestation rates in focus trees during 10 consecutive years. We found increasing seed infestation rates with decreasing host tree densities in years of low seed production, indicating a RDH pattern of seed predators. However, such pattern was not persistent in years of high seed production during which seed infestation rates did not depend on host tree densities. We showed that temporal variations in resource density can lead to transience of seed predator spatial distribution. This study highlights how predictions of plant-herbivore interactions in natural ecosystems may rely on temporal components underlying RCH and RDH hypotheses.

## Introduction

The distribution of organisms is globally driven by spatial availability of limiting resources, foraging capacity and diverse forms of interactions with other species^1–4^. Understanding how foraging efficiency and use of available resources shape organisms’ spatial distribution constitutes one of the ultimate goal of ecology^5^. Developed in the 1960s by behavioural ecologists, the optimal foraging theory provides a conceptual and methodological framework to predict the spatial distribution of organisms with regard to that of their resources^6,7^. The ideal free distribution model (IFD) has been further proposed to infer organisms’ distribution strategies in patchy environments with heterogeneously distributed resources^8^. IFD provided a robust reference model which predictions were supported by empirical and analytical studies on various vertebrate and invertebrate organisms^9–14^. However, deviations from IFD predictions due to underuse of rich sites or overuse of poor sites have been associated with traveling costs^15^, perception limits^16^, intraspecific competition^15,17^ and resource superabundance^18^. These deviations emphasize the need for novel IFD-based predicting approaches^19–21^.

Plant-insect interactions provided critical opportunities to address alternative organism-resource distribution patterns to IFD as the spatial distribution of herbivorous insects is more closely linked to that of their host plant throughout their life than other animal groups with their resources^22–24^. A pioneer article showed that specialist herbivorous insects are more likely to detect and exploit host plants that grow in dense or monospecific stands, leading to the resource concentration hypothesis of herbivores’ distributions (RCH)^25^. Experimental studies have supported RCH predictions in insects^26–28^ but evidence of higher densities of herbivores on isolated or low-density host plants^29,30^ revealed the possibility of opposite distribution patterns that led to the resource dilution hypothesis of herbivores’ distributions (RDH). The prediction of RCH or RDH patterns depends on diverse processes involving mechanistic forces (e.g. senses used for host location and active or passive dispersal mode), energetic pressures (e.g. insect food requirement during its life cycle may imply changing host, mobile or rather immobile feeding stages, diet breadth), or interactions with other animal species (e.g. competition or predation)^31^. In addition, distribution patterns of herbivorous insects may be context-dependent due to other factors associated with neighbourhood effects and scale-dependent regarding the spatial scale at which resource concentration or dilution effects are tested^32–35^. Thus, interdependence between such factors and the specificity of each herbivore-plant study system^36^ make general predictions challenging and support case-by-case or trait-based approaches of herbivore responses to habitat heterogeneity^22^.

One important limitation to the prediction of herbivore distributions is the lack of consideration of temporal components underlying RCH and RDH hypotheses^37,38^. Indeed, plant-insect herbivore interactions likely vary across time according for instance to temporal variation in resource abundance^39,40^. Therefore, taking into account both spatial and temporal dynamics of resource availability is of critical importance to understand herbivore distributions, and more importantly to assess whether observed distributions are transient or persistent when resource distribution display consistent variations that do not result from herbivory itself. Moreover, occurrences of both RCH and RDH patterns have been primarily documented in agricultural crop-insect pest systems^31,41,42^, while knowledge remains limited regarding wild systems such as prairie fields^38,43^ or forests^28,44^. Tree-seed insect systems allow to address these issues. Tree seeds represent a highly variable and unpredictable source of food for insects in both space and time. First, the spatial distribution of seed-producing structures can be heterogeneous within and among tree populations due to individual tree factors and microsite-scale factors^45,46^. Second, at the temporal level, the amount of seed production depends on multiple factors that vary annually among which climate, flowering and pollination rates, recent history of seed production, and density and structure of the tree population^47^. In many tree species, seed production follows an intermittent and synchronous production of large seed crops (‘mast years’) and low to null seed crops ('non-mast years') at the tree population level, a phenomenon referred to as masting^48,49^. Masting generates unpredictable differences in resource density between successive years of seed production which have a pivotal role on predispersal seed predator abundance^46,47,50,51^. The predator satiation hypothesis^48,50^ predicts that overabundance of seeds during mast years tend to satiate seed predators, while seed shortage during non-mast years results in their starvation. Consequently, seed infestation rates are likely to differ between mast and non-mast years^52–55^.

The aim of this study was to assess the impact of temporal variation in resource density, i.e. masting, on the spatial distribution of seed predators in a natural forest ecosystem.

Does the distribution of seed predators follow a global spatial RCH or RDH pattern and does this pattern change according to variations in annual seed production? We used an insect-tree interaction system involving seed wasps (*Megastimus spp*., Hymenoptera: Torymidae) and Atlas cedars (*Cedrus atlantica*., Pinales: Pinaceae), which displays the following appropriate characteristics to address this issue. First, densities of cedar trees vary in natural cedar forests and the species displays a strong synchronized masting pattern of seed production^56^, resulting in spatially and temporally varying patterns of resource density. Second, cedar seed wasps are highly specialized predispersal seed predators whose life cycle and demography are intimately related to any variation in seed abundance on their obligate host^46^. Finally, these wasps have neither other competitors for the seed resource during the predispersal phase nor natural specialist enemies in our study area^57^, which constitutes a simple dynamic system of insect-tree interactions. We conducted a 10-year survey of seed production and seed predator distribution relative to the seed resource (referred to as seed infestation rates hereafter) in focused trees, which occurred in heterogeneous neighbourhood densities in a natural cedar forest of Southern France. We hypothesized that the spatial distribution of seed predators depends on spatial variation in host density (Hypothesis 1, Table 1) and on temporal variation in individual seed production (Hypothesis 2, Table 1), and that such influence of host density can be balanced by temporal variation in individual seed production (Hypothesis 3, Table 1). We predict that seed predators may be prone to follow a RDH or a RCH pattern in years of overall low resource availability within the host population, while their distribution may be less dependent on host density in years of high overall resource density.

**Table 1:** Hypotheses and predictions for the spatial distribution of seed predators in response to spatio-temporal variations in seed resource density within a host population. Seed infestation rates refer to seed predator distribution relative to the seed resource. Neighbourhood density refers to an isolation degree of seed infested trees encompassing both conspecifics’ density and active flight ability of seed predators.

Hypotheses	Predictions	Test	Evidence from this study
Hypothesis 1: Seed predator distribution globally depends on spatial variations in host density	Within population variation in seed infestation rates is explained by neighbourhood density: - seed infestation rates increase when neighbourhood density increases (RCH pattern) - seed infestation rates increase when neighbourhood density decreases (RDH pattern)	Significance and sign of the effect of neighbourhood density on seed infestation rates	Neighbourhood density alone did not explain within population variation in seed infestation rates, i.e. no RCH and RDH patterns were detected
Hypothesis 2: Seed predator distribution globally depends on temporal variations in seed resource	Within population variation in seed infestation rates is explained by seed production	Significance and sign of the effect of seed production on seed infestation rates	Temporal variation in seed resource significantly influenced the within population variation in seed infestation rates
Hypothesis 3: Seed predator distribution depends on both spatial and temporal variations in seed resource	Impact of neighbourhood density on the within population variation in seed infestation rates depends on seed production	Significance and sign of the interaction effect between seed production and neighbourhood density on seed infestation rates	The effect of neighbourhood density on within population seed infestation rates depended on seed production, i.e. a RDH pattern was supported only in non-mast years

## Results

### Temporal patterns of variation in seed production, seed infestation rate and wasp abundance index

The computation of masting metrics based on seed production data supported a strong masting pattern in this cedar population, i.e. high coefficients of variation at both population (*CV*_*p*_ = 1.64) and individual (*CV*_*i*_ = 1.34) levels and a high coefficient of synchrony (*r* = 0.70).

**Figure 1:**
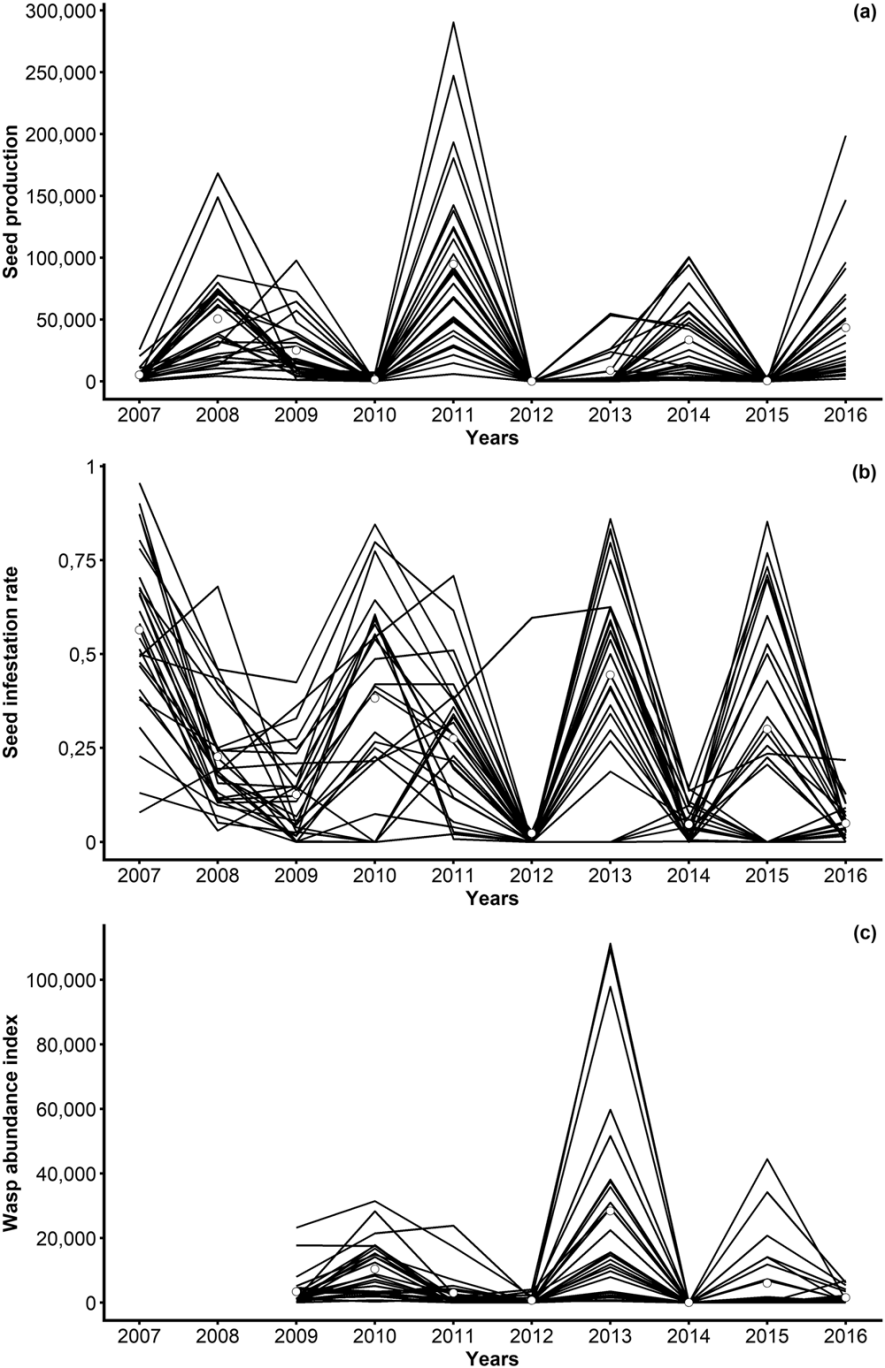
Inter-annual variation in individual seed production (**a**), seed infestation rate by seed wasps (**b**) and wasp abundance index (**c**) in 27 cedar trees at Luberon, France. Lines represent the individual tree values, circles represent the mean values. The wasp abundance index is an estimate of the number of emerging wasps from seeds of each individual tree (computed from 2009 to 2016 as wasps emerged two years after the first seed production estimation in 2007).

The mean seed production exhibited significant inter-annual variation (ANOVA, F = 101.9358, P-value < 2.2 × 10^−16^) (Fig. 1a). Using a non-parametric Duncan test combined with graphical information on seed production data, we categorized 2007, 2010, 2012, 2013 and 2015 as non-mast years and 2008, 2009, 2011, 2014 and 2016 as mast years. Mean seed productions among mast years and non-mast years were of 42 309.82 (SD ± 48 926.187) and 3 169.29 (SD ± 8 124.656) seeds, respectively.

Considering individual tree data, the difference in seed production between mast and non-mast categories of years was highly significant (ANOVA, F = 5.5485, P-value = 5.331 × 10^−14^). For instance, one tree produced an estimated number of 290 325 seeds in 2011 (mast year) and no seeds in 2012 (non-mast year). The lowest seed production over the decade occurred in 2012, with only three trees out of the 27 focus trees producing a total of 46 seeds. These seeds could however not be laboratory-processed for seed infestation rate estimations, which prevented us to use that year from the statistical modelling process of seed infestation rates.

The seed infestation rate, i.e. the distribution pattern of seed wasps relatively to the abundance of resources, varied both between years and between trees (Fig. 1b). The mean seed infestation rate was lower during mast years (0.17, SD ± 0.18) than during non-mast years (0.50, SD ± 0.23).

The wasp abundance index, i.e. the number of wasps emerging from seeds produced by each tree, was also synchronized among trees (Fig. 1c). We observed a trend of negative temporal correlation with a lag of two years between the amount of resources and wasp abundance index (e.g. 2010, 2013 and 2015 had lower seed production and higher wasp abundance index, while 2011 and 2016 showed the opposite pattern), but this was not systematic (e.g. 2012 had low seed production and low wasp abundance index). The mean wasp abundance index among mast years and non-mast years were respectively 1 580.50 (SD ± 3 742.72) and 12 521.17 (SD ± 20 576.72).

### Drivers of spatial distribution patterns of seed wasps relative to their resource

As expected under the predator satiation hypothesis, the model showed that masting significantly decreases seed infestation rate. Furthermore, masting influenced the effect of other spatial and temporal driving factors considered in the model (Table 2). The overall effect of seed production on seed infestation rate was not significant, while this factor slightly but significantly decreased seed infestation rate during mast years only (Table 2 and Supplementary Fig. S1). The effect of the neighbourhood index (i.e. isolation degree of seed infested trees from their conspecifics) nested in masting on seed infestation rate was not significant (Table 2). This showed that local density of conspecific hosts, alone, did not explain seed infestation rate.

**Table 2:** Drivers of cedar seed infestation rate by specialized seed wasps in 27 cedar trees at Luberon (France) over the period 2007–2016. Drivers were inferred from significance of fixed and random effects in the mixed model (see text for definitions). Effect P-values indicate the global significance of the effect. Parameter P-values (fixed effects) refer to the test of null parameter values. Comparison P-values (random effects) inform on the comparison of the two variance estimates. See Table 1 for details on associated hypotheses and predictions.

Hypotheses of Table 1	Fixed Effects	Masting year category	Parameter estimate	Effect P-value	Parameter P-value
	*Masting*			0.009	
		Mast Non-mast	−0.85 0.85		0.008
Hyp. 1	*Masting*/*Neigbh*			0.811	
		Mast Non-mast	−0.06 −0.33		0.632 0.663
Hyp. 2	*Masting*/*Seedprod*			0.096	
		Mast Non-mast	−0.20 0.30		0.032 0.707
	*Masting*/*Wasp*			0.001	
		Mast Non-mast	1.08 0.10		0.000 0.297
Hyp. 3	*Masting*/*Seedprod: Neigbh*			0.005	
		Mast Non-mast	−0.39 −0.29		0.001 0.796
	**Random effects**	**Masting year category**	**Variance estimate**	**Effect** **P-value**	**Comparison** **P-value**
	(0+*Masting*)|*Year*			2.78 × 10^−12^	
		Mast Non-mast	0.47 0.02		0.026
	(0 + *Masting*)|*TreeID*			2.90 × 10^−9^	
		Mast Non-mast	0.35 0.34		1

**Figure 2:**
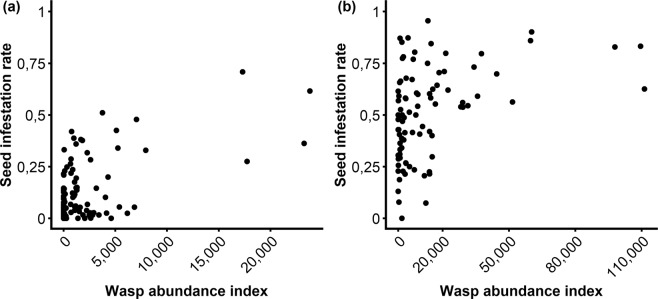
Relationship between seed infestation rate and wasp abundance index during mast (**a**) and non-mast (**b**) years in 27 cedar trees at Luberon, France. Note that the scale of wasp abundance index (*x*-axis) differs between both graphs.

The overall effect of wasp abundance index nested in masting was significant with a significant positive effect during mast years and no effect in non-mast years (Table 2 and Fig. 2).

**Figure 3:**
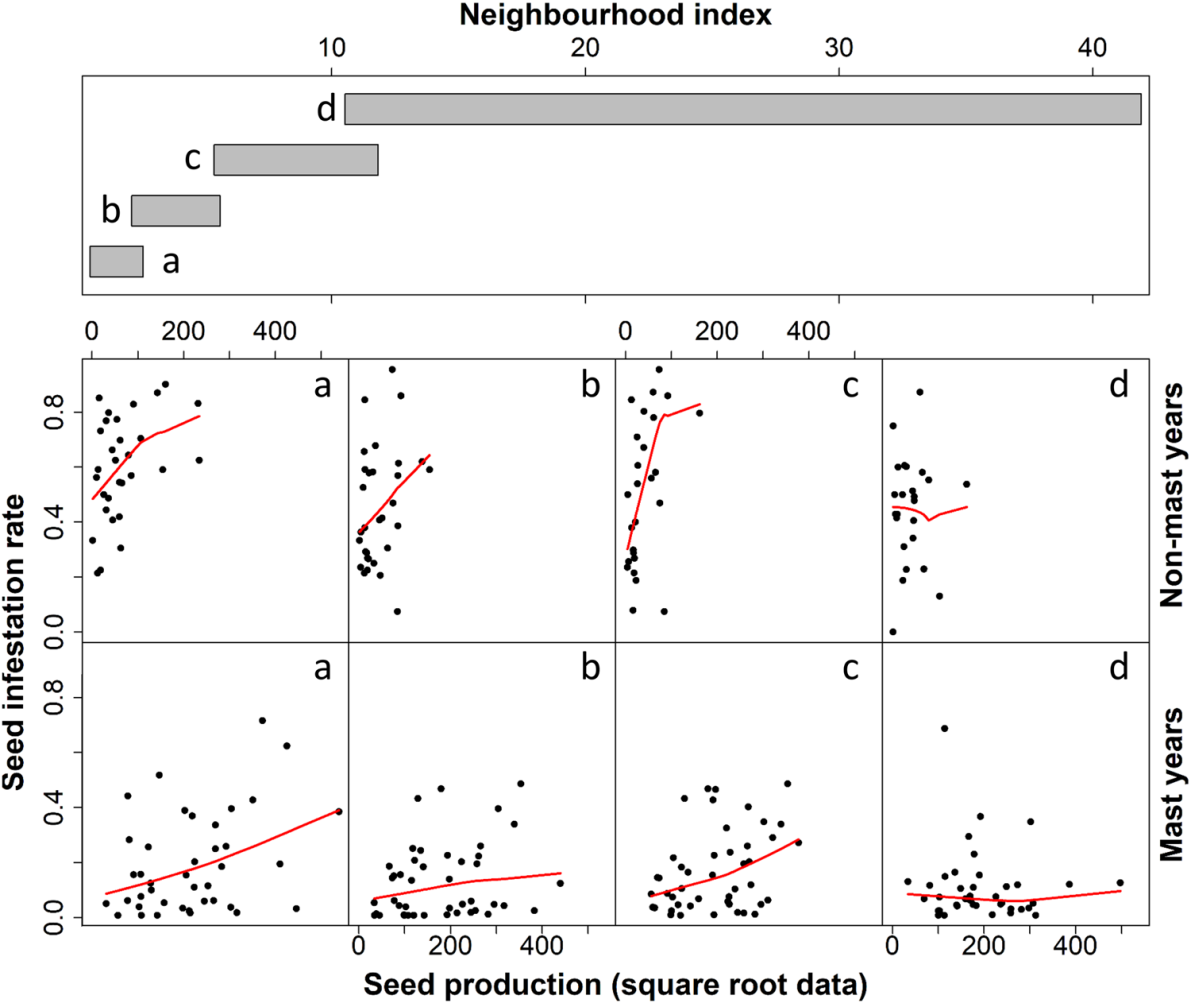
Conditioning plot (Coplot) of variation in seed infestation rate with seed production in relation with neighbourhood density during non-mast years and mast years in 27 cedar trees at Luberon, France. The bars in the top frame shows the division of neighbourhood index into four classes with equal sample size (i.e. **a**: isolated trees, **b**,**c**: two levels of low conspecific density, **d**: high local conspecific density). Red lines are seed infestation rates panel-smooth running means. Note that seed production is presented here as root square transformed data to compensate visually the quantitative discrepancy in seed production between mast and non-mast years. Each point represents a seed infestation rate of a tree in one year.

The model indicated a significant interaction between seed production and neighbourhood index nested in masting (Table 2). We illustrate this complex interaction on Fig. 3. During non-mast years, there was evidence of varying responses of seed infestation rate to seed production depending on neighbourhood indices (Fig. 3). Seed infestation rate increased with seed production only for neighbourhood indices below a value of 10, i.e. isolated trees to trees in low density, and such a trend was supported by significant Pearson correlation coefficients between these variables in this range (Table 3). Conversely, during mast years, we only detected this trend for isolated trees but not for trees in low density (Fig. 3 and Table 3).

**Table 3:** Relationship between seed infestation rate and seed production depending on the density of conspecifics in the nearby environment of seed infested trees (expressed here as a neighbourhood index defining a neighbourhood density, see Fig. 3). This relationship was assessed during non-mast and mast years in 27 cedar trees at Luberon, France. Pearson correlation tests (P-values) were computed in 18 successive windows of 10 individuals along the range of neighbourhood index values.

Median neighbourhood index	Neighbourhood density	P-values	
		Mast-years	Non-Mast years
1.94	Isolated tree	0.001	0.004
1.99	Isolated tree	0.026	0.001
2.12	Isolated tree	0.047	0.001
2.56	Isolated tree	0.043	0.022
2.87	Isolated tree	ns	0.014
2.97	Isolated tree	0.022	0.014
4.92	Low	ns	0.045
4.99	Low	ns	ns
5.38	Low	ns	0.030
5.48	Low	ns	0.038
5.59	Low	ns	0.040
5.63	Low	0.014	0.017
5.77	Low	ns	0.035
6.89	Low	ns	ns
7.59	Low	ns	0.042
10.53	High	ns	ns
11.82	High	ns	ns
12.34	High	ns	ns

### Model random effects

**Figure 4:**
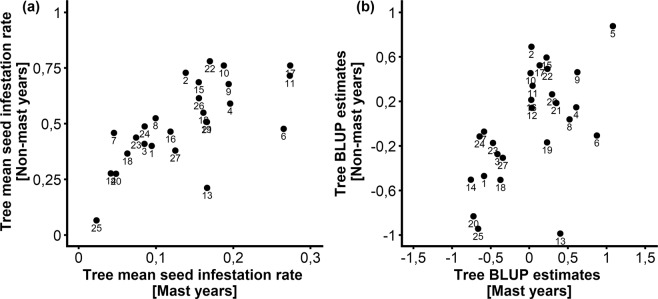
Individual tree effect on seed infestation rate in 27 cedar trees at Luberon, France. (**a**) Relationship between mean observed individual seed infestation rates across mast and non-mast years. (**b**) Relationship between individual best linear unbiased predictors (BLUPs) estimates during mast and non-mast years. Trees are identified by the same number in both graphs (1 to 27).

Both year and tree random effects were significant (Table 2). Inter-annual variance of seed infestation rate was higher during mast years than during non-mast years (Table 2). Inter-individual variance of seed infestation rate was similar between mast and non-mast years (Table 2). Interestingly, the significant positive correlation between mean observed tree-level seed infestation rate in mast and non-mast years (Fig. 4a, Spearman correlation: rho = 0.71, P-value = 4.49 × 10^−5^) was confirmed by a significant positive correlation of tree-level best linear unbiased predictors (BLUPs) estimates (Fig. 4b, Spearman correlation: rho = 0.54, P-value = 0.004). This shows that individual tree characteristics beyond seed production and local neighbourhood drive the seed infestation rate.

## Discussion

One central finding of this 10-year longitudinal survey was that both spatial and temporal patterns of the resource provided by a masting tree population dynamically drive the relative distribution of seed predators.

The masting metrics estimated in our focus cedar population showed high population coefficients of variation and synchrony in seed production that were consistent with other cedar populations^56,58^ and with other tree species displaying strong masting patterns^59,60^. Cedar seeds thus constituted a resource that importantly varied in density at both spatial (among host trees) and temporal scales, and the correlated temporal pattern of seed infestation rate was a clear indicator that resource fluctuation is a key underlying factor of seed predator foraging strategies and spatial distribution pattern within its local host population.

During non-mast years, seed infestation rates reached higher levels than during mast years. This may result from larger abundance of emerging seed predators (favoured by large seed amounts during previous mast years) and the occurrence of a seed resource at lower abundance. One consequence of such resource limitation during non-mast years was an overall number of infested seeds in the population, which led to lower abundance of emerging seed predators and lower seed infestation rates during subsequent mast years. This is in line with the predator satiation hypothesis that predicts alternating periods of seed predator satiation and starvation due to overabundance and shortage of seeds during mast and non-mast years, respectively^48,50^.

We found a pivotal role of masting on both inter-annual and inter-individual variation in seed infestation rates in this cedar population, which resulted in a significant but non-persistent RDH pattern throughout the 10-year study period. Our model showed that considering an interaction between host’s density and pulses in resource availability increased the explanatory power of seed infestation rate compared to host density only. The occurrence of a RDH pattern, i.e. increasing seed infestation rates with decreasing host tree densities, during non-mast years that did not persist during mast-years underlines the critical importance of integrating the potential for resource temporality effects to modulate insect herbivore distributions within local host populations.

We posit that transient distributions of seed infestation rates throughout the study period resulted from different foraging strategies of seed predators between mast and non-mast years. There was no global density effect (cone collection zone) but only micro-environmental neighbourhood effect. During non-mast years, seed production was drastically reduced compared to mast-years, and cedars in higher conspecific density (neighbouring index >10) exhibited lower seed infestation rate with no relationship with seed production, while more isolated cedars (neighbouring index <10) exhibited higher seed infestation rates that increased with increasing seed production. This reflects a crowding effect of seed predators towards isolated individuals, i.e. an increase in seed predator density in low proportion of host areas^61^ that basically characterizes RDH patterns of herbivore distribution. Isolated conifers generally undergo lower intraspecific competition for light that is more beneficial to cone production during both mast and non-mast years than in high-density areas^45^. This may confer higher detectability and attractivity of isolated trees for seed predators in a context of overall low resource availability in the host population. In mobile insects, including seed wasps of this study, host detection for oviposition involves a variety of visual and olfactory cues that may result in preferential foraging bias towards particular hosts among others, e.g. isolated trees^62,63^. The finding of scattered cones within the host population is indeed known to involve short-range dispersal flights in seed wasps^46^, while such foraging flights may be associated with dispersal costs including energy expense and predation risks^64^. Isolated hosts may indeed concentrate specialized predators of insects that would benefit from increased densities of their preys^65^. However, none of the natural enemies of cedar seed wasps in their native range have been detected to date in French cedar populations^66^ which may explain the observed RDH pattern in this study. During mast years, no clear RCH nor RDH patterns were detected as seed infestation rates were not correlated with neighbourhood indices except for isolated trees. This indicates that local resource density did not drive the inter-individual variation in seed infestation rates. As seed wasps likely respond to the same visual and olfactory cues during mast years as during non-mast years, masting may increase the detectability, attractiveness and availability of cone producing trees. This may thus limit their dispersal to random trajectories towards cone producing trees in their neighbourhood whatever tree density. One should however note that some most isolated trees (neighbouring index <4) displayed higher seed infestation rates during both non-mast and mast years, supporting a strong attractiveness of such individuals whatever the overall resource density in the tree population.

This longitudinal study allowed to test for individual tree effect on seed infestation rate which revealed to be significant, beyond the effects of variation in seed production, individual neighbourhood and wasp abundance (included in the model), beyond tree size (that was not kept in the model having no effect), and regardless of masting categories of years. The possible mechanisms underlying such effect is still an open question. Micro-environmental factors other than neighbourhood or tree genetic factors that were not assessed in this study might be responsible for increased or decreased attractivity of particular trees for these seed wasps. Further investigations on other determinants of inter-individual variability in seed infestation rates might be needed.

Overall, this study provides novel support that insect herbivore spatial distributions can be transient in response to temporal variation in resource densities^67^. Our study system also illustrates the benefits of longitudinal studies of plant-insect interactions, especially within natural ecosystems such as forested areas^44,67^ or prairies fields and hills^38,68^ for which the drivers of species spatial distributions in a local environment were still rarely assessed. Masting is a common, but not universal, reproductive strategy of long-lived tree species^48,49^, and it has been widely acknowledged as a strong demographic and evolutionary driving force of seed specialized insects^46,50,51^. In this line, the present study provided critical support for the need to consider seed densities at both tree and populations levels when assessing distributions of insect seed predators^69,70^. Different mechanisms may however arise in other plant-insect interaction systems, depending on whether they involve more generalist insect herbivores, insects with more limited dispersal abilities among tree populations, or important interferences of local neighbouring vegetation with insects’ behaviour. Monophagous and oligophagous predispersal seed predators showed diverging spatial distribution responses to masting patterns in seed production of a jointly exploited perennial herb^68^. Moreover, compared to highly mobile seed wasps, *Curculio* weevil species with low dispersal abilities rather respond to oak masting by aggregating on seed-rich trees^69,70^ or entering prolonged diapause (i.e temporal dispersal) as an alternative strategy to face local resource unpredictability^71^. Finally, the spatial dynamics of insect herbivory may also relate to the relative densities of both host conspecifics and heterospecific neighbours^72^. Such plant associational effects can generate RCH or RDH patterns by either reducing or increasing host use by insects, namely associational resistance^73^ and associational susceptibility^74^ respectively. However, associational effects may not emerge from our study system, where host trees of seed wasps were clearly dominant in both density and size on any other type of vegetation, which mainly consisted of herbs and shrubs in the understorey. A next step to this work will be to test how a spatial scale change might affect the seed wasps’ foraging behaviour, as resource dilution effects can be more important than resource concentration effects on a landscape scale^30,33^.

## Methods

### Study system and study site

In the mid-nineteenth century, the Atlas cedar *C*. *atlantica* was introduced from Northern Africa for the reforestation of degraded lands in South of France where it later expanded by natural regeneration^75,76^. Cone production shows inter-individual variation within populations that may result from individual characteristics (e.g. genotype, age, size, fertilization success), micro-site scale soil and light influences^58,77^. Atlas cedar is considered as a masting tree species exhibiting strong interannual fluctuations of cone production that are relatively well synchronized among trees of a population i.e. both good and less good cone producing trees show variation in cone production in the same year^56^. The amount of cones produced annually by a single cedar tree can vary from zero up to thousands^77^.

In southern France, Atlas cedar is the obligate host of two highly specialized exotic seed wasps, *Megastigmus pinsapinis* and *M*. *schimitscheki* (Hymenoptera: Torymidae), that co-occur during the early stages of cedar cone development. These two *Megastigmus* species are close phylogenetically related^78^,they share the same univoltine life-cycle and respond to the same host clues during their foraging activity^79,80^. In spring, adults emerge from seeds on the ground and females seek for cones to oviposit within new developing seeds on trees. Each larva develops within a single seed at a rate of only one larva per seed and enters at the last developmental stage a two-year period of obligatory developmental arrest (larval diapause) that coincides with the time required for seed maturation and release. The demography of cedar seed wasps is significantly constrained by cedar masting through typical alternating satiation-starvation episodes associated with drastic variations in resource density^66^. During the oviposition period, females use active short-distance flights within a radius not exceeding ca. 20–30 m to forage for available cones in their emerging area^77^ (A. Roques, pers. comm.). Based on such common features between *M*. *pinsapinis* and *M*. *schimitscheki* and the fact that they are the only predispersal seed predators of the Atlas cedar in this area, we analysed cedar seed infestation independently from the species (see further in Methods).

This study was conducted in a natural Atlas cedar forest located in the Petit Luberon Massif in southeastern France (43°47'47.50′′N, 5°14'28.50′′E, 670–700 m.a.s.l.). Original trees were massively planted first in the eastern part of the Petit Luberon Massif ridge ca. 1860 and since then they have extended over a 10 km gradient by natural regeneration towards the West. We focused on 27 cone-producing trees distributed along a transect on this gradient to integrate variation in global tree density resulting from the Westward expansion of this cedar population. During ten consecutive years (2007–2016), each of these focus trees was subjected to an estimation of both cone and seed production and of seed infestation by seed wasp larvae.

Additional individual tree features including dendrometric and neighbourhood characteristics were also assessed for each of the 27 focus trees for the influence they may have on seed wasp foraging activity and seed infestation rates.

### Cedar seed production and individual tree characteristics

In late summer each year, exhaustive counts of mature (two-year-old) cone cohorts were carried out by the same observer in order to limit counting bias using binoculars. In fall each year, we collected a random sample of five mature cones per focus tree prior to seasonal cone disarticulation at two meters above ground. We then disarticulated each cone to separate early aborted seeds that are not targeted by seed wasps from non-aborted ones, which were counted exhaustively. We estimated the total seed production per tree *i* and per year *y* as follows:

1 $$Seedpro{d}_{y,i}=number\,of\,cone{s}_{y,i}\times mean\,number\,of\,non-aborted\,seeds\,per\,con{e}_{y,i}$$

The masting pattern of Atlas cedar was characterized with the following metrics: (i) the population-level inter-annual coefficient of variation of seed production *CVp* computed from the average seed production per trees within the population over time^53^, (ii) the individual-level coefficient of seed production *CVi* across all years using the standard deviations and means of individual tree seed production in 2007–2016^81^, and (iii) the synchrony of seed production among trees using the mean Pearson’s cross-correlation of seed production among all individuals^82,83^.

Individual dendrometrical information on focus trees included measures of diameter at breast height (DBH) and height in July 2011. We assumed that the slight variations in these characteristics over the study period did not have any influence on seed infestation rates. We performed preliminary pairwise correlations tests with the “cor.table” function of the *picante* package^84^ in the R v.3.4.0. statistical software^85^ to identify collinearity among potential explanatory variables and ensure validity in further statistic modelling. We consequently excluded tree height from the analyses as it was highly correlated to DBH measurements (Pearson r > 0.50).

We also aimed at defining a degree of isolation for each focus tree encompassing both conspecifics’ density and distance in relation to the active flight capacities of foraging seed wasps. For this purpose, we recorded all nearby cedar individuals (>2 m height) in a radius of 25 m centred on the focus tree, whether they were cone producers or not as we hypothesized that sterile conspecifics might also act as a visual barrier to seed wasp foraging. We did not consider shrubs (e.g. junipers and boxwoods) and small-sized trees below the canopy of the focus trees (<2 m height, e.g. holm oaks). We measured the distances of nearby conspecifics to the focus tree to compute a neighbourhood index that was adapted from the literature^86,87^ to assess the influence of conspecifics in the nearby environment on seed infestation rate:

2 $$Neigh{b}_{i}=\sum _{j}{e}^{-\propto dij}$$

with *j* the conspecific of focus tree *i*, *dij* the distance from the conspecific *j* to the focus tree *i*, and α a constant coefficient set to 0.1 to align the neighbourhood index with field observations of average active flight within 25 m distance.

### Cedar seed infestation by seed wasps

Seed infestation rates of each cedar individual were used to assess the spatial distribution of seed wasps within the cedar population. Following cone disarticulation and seed extraction, we used numerical X-ray radiography to separate seeds infested by *Megastigmus* larvae from non-infested ones in each focus tree^88^. At this stage it was not possible to differentiate between the two wasp species, but as there is always one larvae per seed^56^, we then computed yearly seed infestation rate for each focus tree independently from the species as follows:

3 $$Seed\,infestation\,rat{e}_{y,i}=number\,of\,infested\,seed{s}_{y,i}/number\,of\,seed{s}_{y,i}$$

Based on individual seed infestation rate estimation, we computed for each focus tree a wasp abundance index as a proxy of the number of adult wasps emerging each year from its own infested seeds as follows:

4 $$Was{p}_{y,i}=\,Seed\,infestation\,rat{e}_{y-2,i}\times Seedpro{d}_{y-2,i}\times D$$

with *y* the year of adult emergence considering that seed infestation occurred two years before due to the two-year cone maturation period, and *D* a constant mortality coefficient of 0.36^66^ applied to each larval cohort across the study period.

### Data analyses

We first divided the study period into two categories of years depending on the amount of seeds produced at population level, namely the mast or the non-mast categories (years of higher and lower seed production, respectively).To assign years to these categories, we combined graphical information on individual seed production data (Fig. 1) and a multiple comparison procedure of seed production between all years with Duncan post-hoc comparison tests using the R *agricolae* package^89^.

The statistical modelling of the response variable i.e. seed infestation rate of year *y* measured on tree *i* was based on a generalized linear mixed model (GLMM) implemented in the R *glmmADMB* package^90,91^. We modelled seed infestation rate, as a proportion, with a logit link function. Using first a model with a binomial family error term, we observed overdispersion in our data with a ratio between residual sum of squares and residual degrees of freedom equal to 8. We thus estimated model parameters using a Beta-Binomial model^92,93^.

We introduced a fixed effect (*Masting*) to account for the pivotal role of categories of years on seed infestation rate into a mixed effect nested model, where all covariates and factors were nested within this effect. We considered the following temporal drivers of the distribution of seed infestation rate: masting year category (*Masting*) as a fixed effect and year as a random effect within *Masting* to account for inter-annual variances within each category. To account for spatial drivers, we considered tree level covariates (tree diameter, seed production and wasp abundance index), individual tree random effect within *Masting* to account for inter-individual variances within each category and micro environmental factors and covariates (cone collection zone and neighbourhood index). All covariates were standardized prior to modelling.

We applied an automated selection of fixed effects using the “dredge” and “get.model” functions in the R *MuMin* package^94^. We used the Aikake Information Criteria corrected for small sample size (AICc) to choose the most parsimonious model providing the best fit to the data^95,96^. We discarded models displaying a ΔAICc < 6 with the most parsimonious one^97–99^ (Supplementary Table S1). Thus, tree diameter and cone collection zone were not kept in the final model, which wrote as follows:

5 $$\begin{array}{rcl}Seed\,infestation\,rate{}_{y,i} & = & \mu +Masting+Masting:Seedpro{d}_{y,i}\\  &  & +Masting:Neigh{b}_{i}+Masting:(Seedpro{d}_{y,i}:Neigh{b}_{i})\\  &  & +Masting:Was{p}_{y,i}+(0+Masting)|TreeI{D}_{i}\\  &  & +(0+Masting)|Yea{r}_{y}+{E}_{y,i}\end{array}$$

where $\mu$ is the overall intercept, and *Masting*, *Seedprod*, *Neighb* and *Wasp* as previously defined in Eqs. (1),(2) and (4). Additionally, random factors *TreeID* and *Year* represent the inter-individual and inter-annual variances, respectively, and were also nested within *Masting* with no variation on the intercept, to allow variance estimates to vary between mast and non-mast categories of years.

We tested the significance of fixed effects with a Fisher test and type III sum of square using the “Anova“ function implemented in the R *car* package^100^. We tested the significance of random effects by model comparisons using likelihood ratio tests (LRT) with the R “anova” function (Supplementary Table S2).

## Supplementary information

Supplementary figures and tables

## Data Availability

INRA has an open-data policy and, once publication decision is taken, the dataset will be available on our institutional archive https://data.inra.fr/.
