# Supplementary material for: Spatial and temporal patterns of a pulsed resource dynamically drive the distribution of specialist herbivores
Source: Sci Rep. 2019 Nov 28;9:17787. doi: 10.1038/s41598-019-54297-6 (PMC6882897; doi:10.1038/s41598-019-54297-6)
Supplement: Supplementary file 1 — Supplementary figures and tables [file 41598_2019_54297_MOESM1_ESM.pdf]

Spatial and temporal patterns of a pulsed resource dynamically drive the distribution of specialist herbivores

Violette DOUBLET[1,*], Cindy GIDOIN[2], François LEFEVRE[1], Thomas BOIVIN[1]

[1]UR 629 Recherches Forestières Méditerranéennes, INRA, 84 914 Avignon Cedex 09, France

[2]Department of Evolutionary Biology and Environmental Studies, University of Zurich, Winterthurerstrasse 190, CH-8057 Zurich, Switzerland

[*] doublet.violette@gmail.com

Supplementary material

Fixed-effects considered	*df*	Log likelihood	AIC	ΔAIC_c
Intercept+	1	-2501.43	5030.86	2.38
Masting +	1			
Masting : Seedprod +	2			
Masting : Neighb +	2			
Masting : Wasp +	2			
Masting : Seedprod: Neighb	2			
Intercept+	1	-2497.09	5030.18	1.70
Masting +	1			
Masting : Seedprod +	2			
Masting : Neighb +	2			
Masting: DBH+	2			
Masting: Cone collection zone +	2			
Masting : Wasp +	2			
Masting : Seedprod : Neighb	2			
Intercept+	1	-2500.49	5032.98	4.50
Masting +	1			
Masting : Seedprod +	2			
Masting : Neighb +	2			
Masting: DBH+	2			
Masting : Wasp +	2			
Masting : Seedprod : Neighb	2			
Intercept+	1	-2498.24	5028.48	0
Masting +	1			
Masting : Seedprod +	2			
Masting : Neighb +	2			
Masting: Cone collection zone +	2			
Masting : Wasp +	2			
Masting : Seedprod: Neighb	2			

Supplementary Table S1. Fixed effects model selection. In these models, *TreeID* and *Year* are included as random factor effects (not shown here). Models are ranked according to Akaike's information criterion adjusted for small sample size (AICc). ΔAIC_c correspond to AIC_{ci} – minimum AIC_c.

Model	Log likelihood	AIC	ΔAIC_c
**No random effect**	-1071.0	2164	64.96
1| TreeID	-1055.9	2135.86	36.82
1| Year	-1055.8	2135.64	36.60
0+Masting| TreeID	-1061.1	2148.26	49.22
0+Masting|Year	-1054.2	2134.36	35.32
(0+Masting|Year)+ (0+Masting|TreeID)	-1034.5	2099.04	0

Supplementary Table S2. Random effects model selection. The model with no random effect is the "full" model selected (all fixed factors and covariates included). Models are ranked according to Akaike's information criterion adjusted for small sample size (AICc). ΔAIC_c correspond to AIC_{ci} – minimum AIC_c.

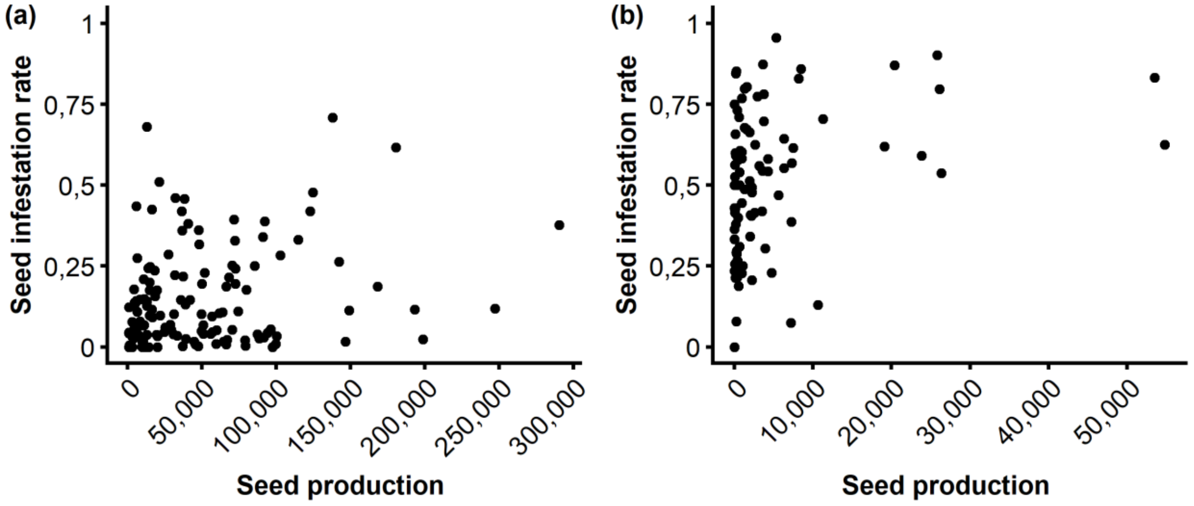

Supplementary Figure S1. Relationship between seed infestation rate and seed production during mast (**a**) and non-mast (**b**) years in 27 trees of the Luberon *Cedrus atlantica* forest, France. Note that x-axis between both graphs are not on the same scale.
